# Proactive iron supplementation alone in patients on chronic hemodialysis is not sufficient

**DOI:** 10.1080/0886022X.2026.2694788

**Published:** 2026-08-05

**Authors:** Panna Pribelszki, Máté Szász, Mihály Tapolyai

**Affiliations:** ^a^ Department of Pharmacy, Szent Margit Hospital, Budapest, Hungary; ^b^ Department of Nephrology, Szent Margit Hospital, Budapest, Hungary; ^c^ Medical University of South, Charleston, SC, USA

**Keywords:** Anemia, iron supplementation, hemodialysis, iron saturation, hemoglobin

## Abstract

Dialysis patients often become iron-deficient because of many factors, including poor iron absorption from the gut, blood loss during dialysis, frequent blood draws, phosphate binders, and the use of erythropoietin. Oral supplementation is ineffective because of elevated hepcidin levels and low levels of iron-absorbing factors in the duodenal mucosa. Intravenous iron supplementation can be given in two forms: one is to replace iron when the iron content or total iron binding capacity saturation (FeSat) is too low, that is, a reactive dosing (RE); or by administering iron on a schedule of weekly proactive dosing (PRO), as recommended by guidelines. We investigated whether PRO alone is sufficient, as our hospital-based dialysis unit used a PRO schedule according to our current protocol. The data of 102 patients receiving a mean 107.7 ± 65.9 mg/week of intravenous iron (sodium ferric gluconate complex) were analyzed. Fifty-four of the 91 patients with complete datasets had an FeSat 25 (*p* = 0.0003). After 3 months of both PRO and RE dosing the mean ± standard deviation hemoglobin rose from 10.07 g/dL ±1.35 with PRO alone to 10.55 ± 1.70 (p:0.0008) using both schedules from FeSat: 24.2% ±14.1 and to 29.22% ±9.8 (p:0.0015). Thus, we conclude that the PRO regimen alone is not sufficient to maintain a healthy hemoglobin or FeSat level while using the same amount of erythropoietin.

## Introduction

The recently published 2026 Kidney Disease [1]: Improving Global Outcomes. The Clinical Practice Guidelines for the Management of Anemia in Chronic Kidney Disease (CKD) have changed previous recommendations regarding iron replacement strategies for CKD in recommending a proactive approach rather than relying solely on a reactive practice pattern.

Iron deficiency is one of the most frequent complications in patients with end-stage kidney disease (ESKD) treated with chronic hemodialysis. Contributing factors include impaired gastrointestinal iron absorption, recurrent blood loss during dialysis procedures, frequent laboratory phlebotomy, and iron-mobilizing effects of erythropoiesis-stimulating agents (ESAs). Adequate iron stores are fundamental for optimizing erythropoiesis and reducing ESA requirements, as stated by Kidney Disease Improving Global Outcomes (KDIGO) and other nephrology guidelines.

Two main approaches exist for intravenous (IV) iron therapy in dialysis units: Proactive iron dosing (PRO): low-dose iron continuously (e.g., 62.5–125 mg/week) administered weekly unless polycythemic or had a total iron binding capacity (TIBC) saturation >45%. Alternatively, the reactive iron dosing (RE), where IV iron is administered when the patient requires it, when the TIBC saturation falls below 25%, 1000 mg iron sucrose given in divided doses.

The landmark PIVOTAL trial [2] (Proactive IV irOn Therapy in hemodiALysis patients (PIVOTAL) trial) demonstrated that a PRO high-dose iron regimen led to better hemoglobin levels, reduced ESA use, and fewer cardiovascular events than the RE strategy. This strategy was idealized in the subsequent guidelines and recommendations. However, many dialysis units still use low-dose proactive regimens, sometimes exclusively, and the efficacy of these minimalist proactive-only strategies remains uncertain.

Our hospital’s dialysis unit employed a proactive-only protocol with low weekly doses of IV sodium ferric gluconate without a reactive treatment strategy for low saturations. We conducted a retrospective cross-sectional analysis to determine whether this regimen alone is sufficient to maintain hemoglobin (Hgb) and iron indices and to compare outcomes with PRO and RE combined treatment.

## Methods

This was a cross-sectional study conducted at the dialysis unit of a community medical center, which is a teaching hospital for the Semmelweis University School of Medicine, Budapest, Hungary. The study was approved by the Institutional Review Board (#IG1909-2/2025) of Szent Margit Hospital, Budapest, and considering the retrospective, nonintervention nature of the study with no identifiable information being used, informed consent waiver was given. Data were obtained from existing patient records of routine monthly dialysis blood tests. No blood tests were performed for the purpose of this study. Laboratory data were de-identified and only aggregate data were analyzed; patient privacy was protected, and the Declaration of Helsinki regarding medical research on human subjects was observed.

Iron studies were drawn with monthly labs, 1 week ‘iron break’ was given prior to blood draws that included iron studies. Iron studies were performed every 3 months in this dialysis unit. In the proactive dosing schedule (PRO), all patients received a maintenance dose of iron (e.g.: 62.5 mg iron sucrose weekly) unless they were polycythemic or had a TIBC saturation >45%. Patients’ iron dosing, who had very low-iron saturation (<25%), was increased to 125 mg of iron sucrose weekly. No RE protocol was in use for those who did not achieve satisfactory iron repletion. However, a reactive iron dosing (RE) was introduced after these data were obtained in addition to the PRO regimen: when the iron saturation was noted to fall below 25%, then 1000 mg of iron sucrose was administered in divided doses: eight doses of iron sucrose 125 mg administered each successive dialysis session, and then, a weekly PRO regimen was restarted. Three months later, the data were re-analyzed.

Statistical analysis for column statistics, chi square, and paired Student’s t-test was performed using Prism statistical software (GraphPad Software, Inc. ver. 5.04) for Windows.

## Results

We had 102 prevalent HD patients whose data were potentially available for analysis; data from 91 patients were analyzed for the PRO group. For the combined PRO+RE group, the data of 11 patients were incomplete and were thus excluded. Our patients were 67.0 ± 13.3 years old (mean ± standard deviation) and 61.7% of them men; 20.2% diabetic. Patients’ pre-dialysis urea was 23.2 ± 6.9 mmol/L and delivered Kt/V: 1.26 ± 0.43. Their serum albumin: 3.74 ± 2.8 mg/dL, i-PTH: 351.4 pg/*L* ± 374.8, CRP: 14.3 ± 26.0 mg/L, and hemoglobin: 10.0 ± 1.3 g/dL.

With this PRO regimen, 56.9% of patients did not reach 25% saturation, and the mean saturation of TIBC was 24.18% ±14.09 (CI: 21.26–27.10) for the entire cohort; median saturation was 22.11%. When the PRO regimen was complemented by a reactive (RE) iron supplementation regimen, 3 months later, the iron parameters were reexamined, and the mean saturation rose to 29.22% ±9.86 (CI: 27.12–31.32) with a median saturation of 28.61%, two-tailed paired t-test *p* = 0.0015. The PRO protocol alone achieved TIBC saturation of >25% in 37 patients and <25% in 54 patients. Combining the PRO and RE protocols, we were able to achieve >25%, saturation in 56 patients, and only 27 patients remained iron deficient of the 83 patients who had data from both protocols; chi square *p* = 0.0003. Hemoglobin values also rose from 10.07 ± 1.35 g/dL (9.79–10.34) to 10.55 ± 1.40 g/dL (10.25–10.84) p: 0.0008 even though the erythropoietin dose did not change (13,516 ± 7126 IU vs. 13,360 ± 8243 IU p: NS).

## Discussion

**Figure 1.**
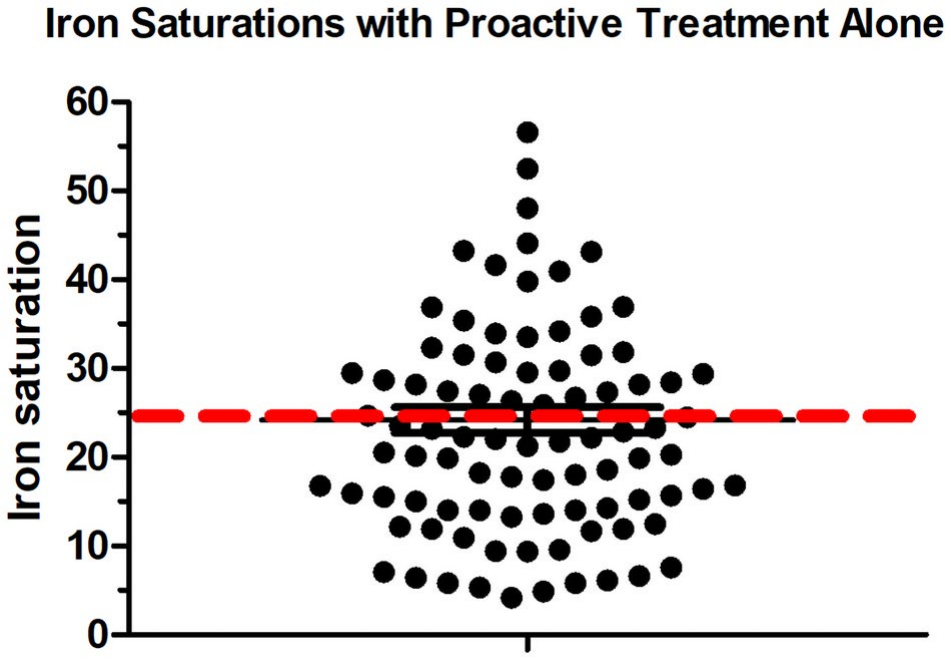
Distribution of total iron binding capacity iron saturations (mean, ±standard deviation) while using exclusively the proactive treatment strategy. 56.9% of patients did not reach 25% saturation, represented by the red line. Median 22.11 Mean 24.18 ± 14.09 (CI: 21.26–27.10).

**Figure 2.**
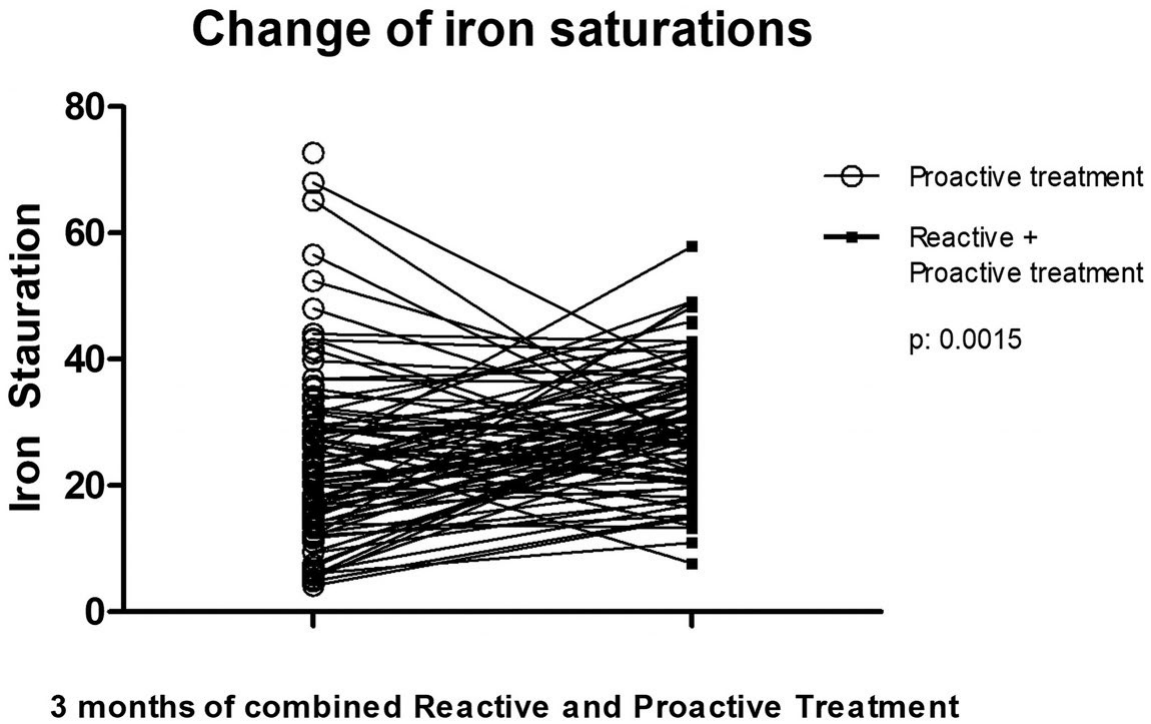
Changes in iron saturations (FeSat) using the proactive treatment strategy then one month of combined proactive and reactive treatment strategies. Paired t-test FeSat: *p* value: 0.0015.

**Figure 3.**
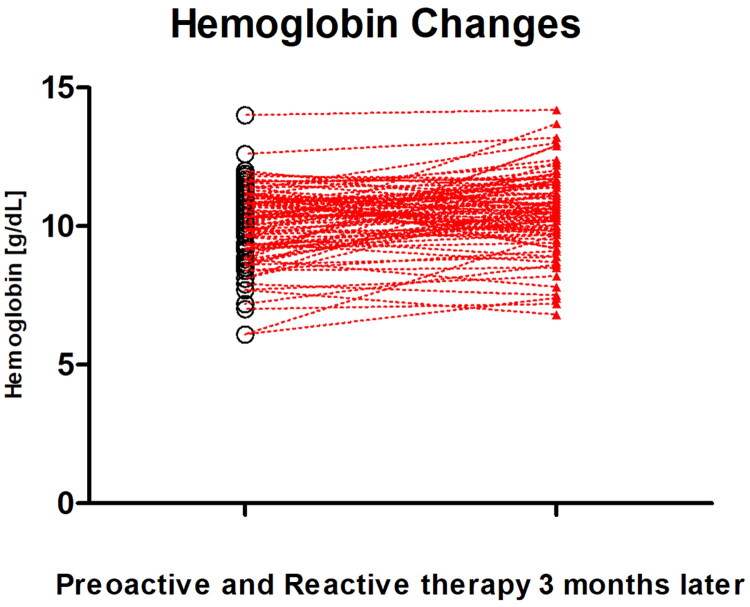
Changes in hemoglobin content using the proactive treatment strategy then one month of combined proactive and reactive treatment strategies. Two tailed paired *t*-test *p*: 0.0008.

The Kidney Disease Improving Global Outcomes (KDIGO) 2026 edition [1], in contrast to the previous 2012 edition [3], recommends that ‘In people with CKD G5HD in whom iron therapy is being initiated, administer i.v. iron using a proactive approach to maintain stable iron status’ under practice point 2.1 as a treatment strategy. This strategy is to administer a low dose, indicating about 100 mg iron per week, iron on a weekly basis to avoid high peak serum iron levels and avoid iron deficiency at the same time. The European Renal Association also endorses such a treatment approach: ‘…we support the proactive administration of IV iron rather than a reactive approach (Practice Point 2.1)’ [4]. Our patients also received this regimen, receiving 107.7 ± 65.9 mg/week on the average. However, patients were not able to maintain an iron-replete state, as evidenced by the cohort’s mean TIBC saturation of 24.18% ±14.09, although more telling that 56.9% of patients did not achieve the desired minimum 25% saturation (Figure 1). When we combined the PRO regimen with the RE strategy, the mean iron saturation increased to 29.22% ±9.86 (*p* = 0.0015) (Figure 2). together with the mean hemoglobins, from 10.07 ± 1.35 g/dL to 10.55 ± 1.40 g/dL (p: 0.0008) (Figure 3) as expected, even though the ESA dose did not change. The local guidelines by the Hungarian Society of Nephrology (MANET) recommend [5] a combination of treatments if the PRO treatment strategy is insufficient; the KDIGO 2026 guidelines do not mention the possible combination, despite our data demonstrating that PRO strategy alone is not enough to maintain an iron replete status. The aim of this study was not to demonstrate how the combination of PRO and RE strategies works simply to show and validate our claim that the PRO strategy alone is not sufficient.

Maintaining iron sufficiency is critical, however, not only for maintaining a healthy hemoglobin level [6] and oxygen transport, but also for mitochondrial function, myocardial contractility [7,8], and exercise tolerance. Additionally, adequate intravenous iron supplementation can reduce the mean dose of ESA requirements, thus making anemia treatment more economical [9].

Notwithstanding, our purpose was simply to highlight that PRO intravenous iron supplementation without the reactive treatment regimen when required in dialysis patients is not sufficient for a stable and acceptable iron saturation level. Therefore, a combination of treatment strategies should be considered.

## Data Availability

The data that support the findings of this study are available from the corresponding author, [MT], upon reasonable request. Basic, Share upon Request

## Accountability statement

All authors agree to be accountable for all aspects of the work.

## References

[1] Eknoyan G, Lameire N, Winkelmayer WC, et al. Kidney disease: improving global outcomes. Clinical Practice Guideline for the Management of Anemia in Chronic Kidney Disease (CKD) Kidney International. 2026;109: s 1–S99. (Suppl 1S), 10.1016/j.kint.2025.06.006 41485812

[2] Macdougall IC, White C, Anker SD, et al. Intravenous iron in patients undergoing maintenance hemodialysis. N Engl J Med. 2019;380(5):447–458. Epub 2018 Oct 26. doi: 10.1056/NEJMoa1810742. 30365356

[3] KDIGO Clinical Practice Guideline for Anemia in Chronic Kidney Disease Kidney International Vol 2 Issue 4 August (2); 2012

[4] Del Vecchio L, Cases A, Eisenga MF, et al. KDIGO 2026 clinical practice guideline for anemia in chronic kidney disease (CKD): a commentary from the European Renal Best Practice (ERBP). Nephrol Dial Transplant. 2026:gfag014. doi: 10.1093/ndt/gfag014. PMC13423824 41604211

[5] Erzsébet Ladányi E, Reusz G. Hypertonia és Nephrologia. Suppl. 1) A Korszerű Dialíziskezelés Gyakorlata. 2021;25.

[6] Camaschella C. Iron-deficiency anemia. N Engl J Med. 2015;372(19):1832–1843. doi: 10.1056/NEJMra1401038. 25946282

[7] Musallam KM, Taher AT. Iron deficiency beyond erythropoiesis: should we be concerned? Curr Med Res Opin. 2018;34(1):81–93. Epub 2017 Nov 3. doi: 10.1080/03007995.2017.1394833. 29050512

[8] RJ, Mentz, J, Garg, FW, Rockhold the HEART-FID Investigators., et al. Ferric carboxymaltose in heart failure with iron deficiency. N Engl J Med. 2023;389(11):975–986. +. doi: 10.1056/NEJMoa2304968. 37632463

[9] Zununi Vahed S, Ahmadian E, Hejazian SM, et al. The impact of intravenous iron supplementation on hematinic parameters and erythropoietin requirements in hemodialysis patients. Adv Ther. 2021;38(8):4413–4424. doi: 10.1007/s12325-021-01826-3. 34254256

